# Pyruvate Kinase M2 and Cancer: The Role of PKM2 in Promoting Tumorigenesis

**DOI:** 10.3389/fonc.2020.00159

**Published:** 2020-03-02

**Authors:** Kulsoom Zahra, Tulika Dey, Ashish, Surendra Pratap Mishra, Uma Pandey

**Affiliations:** ^1^Department of Biochemistry, Institute of Medical Sciences, Banaras Hindu University, Varanasi, India; ^2^Department of Anatomy, Institute of Medical Sciences, Banaras Hindu University, Varanasi, India; ^3^Department of Obstetrics and Gynecology, Institute of Medical Sciences, Banaras Hindu University, Varanasi, India

**Keywords:** pyruvate kinase M2, anaerobic glycolysis, angiogenesis, chemotherapy, cancer metabolism

## Abstract

Pyruvate kinase plays a pivotal role in regulating cell metabolism. The final and rate-limiting step of glycolysis is the conversion of Phosphoenolpyruvate (PEP) to Pyruvate, which is catalyzed by Pyruvate Kinase. There are four isomeric, tissue-specific forms of Pyruvate Kinase found in mammals: PKL, PKR, PKM1, and PKM2. PKM1 and PKM2 are formed bya single mRNA transcript of the PKM gene by alternative splicing. The oligomers of PKM2 exist in high activity tetramer and low activity dimer forms. The dimer PKM2 regulates the rate-limiting step of glycolysis that shifts the glucose metabolism from the normal respiratory chain to lactate production in tumor cells. Besides its role as a metabolic regulator, it also acts as protein kinase, which contributes to tumorigenesis. This review is focused on the metabolic role of pyruvate kinase M2 in normal cells vs. cancerous cells and its regulation at the transcriptional level. The review also highlights the role of PKM2 as a potential diagnostic marker and as a therapeutic target in cancer treatment.

## Introduction

**Figure 1:**
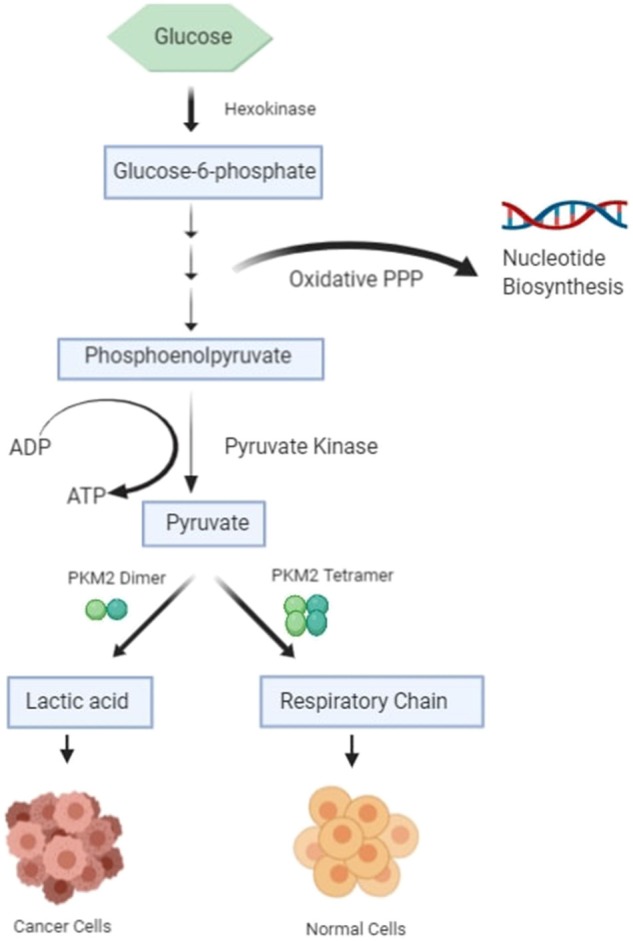
Schematic diagram of glucose metabolism in healthy cells and cancer cells.

The most important hallmark of the cancer cell is metabolic reprogramming. Tumor cells are different from healthy cells in the sense that they are dependent on aerobic glycolysis to produce energy even if there is sufficient oxygen in the surroundings (1–3). This switching of the cell from the normal respiratory pathway to aerobic glycolysis is known as the Warburg effect (1–3). The cancer cell produces additional energy by increasing the reaction rate of glycolysis with the production of lactate in cytosol (4). In healthy cells, pyruvate is either wholly oxidized to CO_2_ with more production of ATP in the presence of oxygen through the mitochondrial respiratory chain (the TCA cycle) or converted to form lactic acid in oxygen-deficient conditions (Figure 1) (4). Pyruvate kinase catalyzes the final reaction of glycolysis, in which the high-energy phosphate group is transferred from phosphoenolpyruvate (PEP) to ADP to form Pyruvate, with the production of ATP (5, 6). There are four types of isomers of Pyruvate Kinase in mammals, PKM1, PKM2, PKL, and PKR; all are tissue-specific, as shown in Table 1 (6). PKR is predominantly expressed in red blood cells, whereas PKL is expressed exclusively in the liver, kidney, and intestine and has the lowest propensity for PEP (6). PKM1 is found to be up-regulated in the tissues that demand a massive supply of energy like the heart, brain, and muscle; PKM2 is expressed in all proliferating cells, especially tumor, and embryonic tissues (7–9). PKM1 and PKM2 are derived from a single PKM gene by alternative splicing of a primary mRNA transcript that contains exon 9 and exon 10, respectively (10). PKM2 is exclusively expressed by the embryonic cells, and as the embryogenesis proceeds, the PKM2 is replaced by tissue-specific PKM1, PKL, or PKR (6). In the case of cancerous cells, PKM2 expression is up-regulated, whereas the expression of tissue-specific PKM1, PKL, and PKR gradually declines (6, 9). PKM1 and PKM2 have different enzymatic properties, and both are regulated by several factors at different transcriptional and post-transcriptional levels. PKM1 is a constitutively active enzyme that shows increased affinity for its substrate PEP, whereas PKM2 enzyme activity is subjected to complex allosteric regulation, which is crucial for tumor progression and growth (4, 11). The expression of PKM2 is up-regulated in most of the cancer cells, suggesting that PKM2 serves as a promising target for cancer treatment (12).

**Table 1:** Tissue-specific isoforms of pyruvate kinase.

**PKL**	**PKR**	**PKM1**	**PKM2**
•Hepatocytes •Intestine •Pancreas •Kidney	•Erythrocytes	•Heart •Skeletal muscle •Smooth muscle •Brain •Adrenal medulla •Mature sperm	•Tumor cells •Embryonic tissue •Testis •Ovaries •Adrenal cortex •Pancreatic islet cells •Thymus •Kidney distal tubule cells •Leucocytes

PKM2 protein switches between a high-activity tetramer form and a low-activity dimer form in healthy tissues (8, 13, 14). However, in tumor cells, it tends to exist as dimer PKM2, which has low catalytic activity, resulting in the increased anabolic synthesis of macromolecules through the PPP pathway and thus promotes cancer cell proliferation and growth (15, 16). As well as its role in metabolic reprogramming, PKM2 also serves as a cytosolic receptor for thyroid hormone (17). It is also involved in the epigenetic regulation of gene transcription (18–20); it enters into the nucleus upon oncogenic stimulation, where it acts as a protein kinase and phosphorylates proteins, including histones (21, 22). However, the role of PKM2 in the regulation of cancer is not entirely understood. This review highlights the role of PKM2 in cancer metastasis, its regulation, and how it could serve as a potential diagnostic marker and therapeutic target in cancer.

## Active and Inactive Isomeric Pyruvate Kinase M2

**Figure 2:**
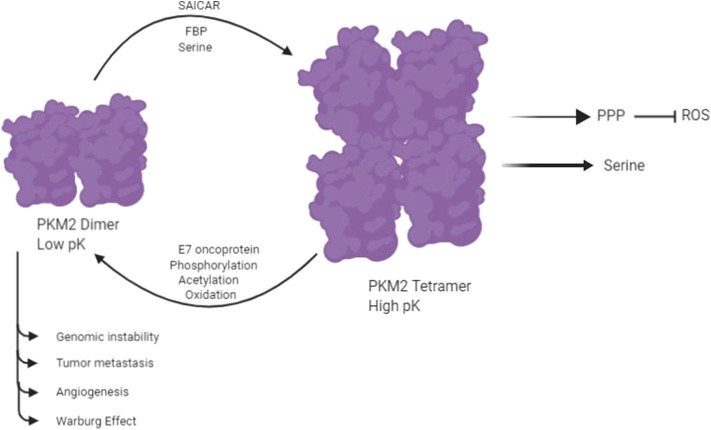
PKM2 exists in two isomeric forms: a highly active tetramer and a low-activity dimer, whereas PKM1 constitutively exists only as a highly active tetramer. Several molecules control the switching between the dimeric and tetrameric forms of PKM2. E7 oncoproteins, tyrosine kinase-mediated phosphorylation, acetylation, and oxidation encourage the formation of low-activity dimer PKM2. In contrast, fructose-1,6-P2, serine, and SAICAR promote the formation of highly active tetramer (18).

Pyruvate Kinase isozymes type PKM1, PKL, and PKR exist instable and high-activity tetramer forms, whereas PKM2 is found in both a highly active tetramer form and a low-activity dimer form (6, 23). PKM2 plays a vital role in glycolysis, and it catalyzes the conversion of phosphoenolpyruvate (PEP) to pyruvate with the production of ATP in the final reaction of glycolysis. PKM2 provides an *in vivo* growth advantage in cancer cells by its preferential expression and allosteric enzymatic activity without accumulation of ROS (24). The switching between the high-activity and low-activity states of PKM2 is subjected to allosteric regulation. The enhanced catalytic activity of tetrameric PKM2 is related to increased production of ATP and catabolism of glycolytic intermediates in a normal cell. The low catalytic activity of dimeric PKM2 results in increased production of glycolytic intermediates by inducing other glycolytic enzymes of the pentose phosphate pathway and glycerol synthesis and producing NADPH, which suppresses ROS production (24, 25). A number of molecules have been reported to be involved in the allosteric regulation of dimer and tetramer PKM2 (Figure 2) (18). An up-stream glycolytic intermediate and an activator of PKM2, fructose 2,3-bisphosphate (FBP), is involved in allosteric regulation of dimer and tetramer PKM2 (26). FBP helps in the formation of the active tetrameric form of PKM2 by binding to its allosteric site (26). PKM2 is also regulated by serine, which binds to PKM2 and activates it, and reduction in the level of serine also reduces the catalytic activity of PKM2 in the cell (27). PKM2 activity is also subjected to regulation by Phosphoribosyl amino imidazole succinocarboxamide (SAICAR), an intermediate of the purine synthesis pathway (13, 14). It allows PKM2 to act as pyruvate kinase as well as protein kinase (13, 14). Interaction of SAICAR with PKM2 allows tumor cells to thrive in glucose-limited conditions (13, 14). PKM2 is subjected to phosphorylation on Y-105, resulting in the release of FBP, which causes switching of PKM2 from the tetramer to the dimer state (3, 28). This allosteric regulation of pyruvate kinase leads to tumor cells that coordinate various metabolic pathways needed for cellular proliferation in nutrient-limited conditions. PKM2 is also subjected to several post-translation modifications like phosphorylation, acetylation, sumoylation, hydroxylation, and oxidation, which prefer the formation of dimer PKM2 in tumor cells (13, 14).

## Regulation of Pyruvate Kinase M2 Gene

The expression PKM2 is regulated by several factors, including heterogeneous ribonucleoproteins (hnRNPs), namely hnRNP1, hnRNP2, and hnRNP3. These factors bind to the PKM gene and initiate alternative splicing of the transcript of an intronic sequence of exon 9 containing PKM1 and further repress its splicing and activate exon 10 of PKM, containing PKM2, which results in the up-regulation of PKM2 mRNA expression (19, 26, 29). PKM2 expression is also up-regulated by Never in mitosis gene A-related kinase 2 (NEK2). NEK2 combines with hnRNP1 or hnRNP2 and promotes the release of exon 10 and activates PKM2. The silencing of NEK2 down-regulates the PKM2 expression (2).

Several non-coding RNAs also regulate the expression of PKM, including miR-148a, miR-152, miR-199a, miR-222, miR-138, and let-7a. These non-coding RNAs directly bind to the 3′ Untranslated Region (UTR) of the PKM2 mRNA transcript and decrease the synthesis of PKM2 protein (20, 30, 31). Besides, c-Myc protein regulates the PKM2 expression in tumor cells. c-Myc activates the transcription of hnRNPs, which directly interacts with exon 9 and suppresses it and allows the simultaneous expression of the PKM2 isoforms (16). Sun et al. experimentally demonstrated that the expression of PKM2 is induced by activated mTOR, a key activator of the Warburg effect in cancer cells. mTOR activates hypoxia-inducible factor 1 (HIF-1) and initiates the c-Myc-hnRNPs–mediated alternative splicing, which leads to aerobic glycolysis in cancer cells (16, 32, 33). In most of the cancer cells, the PI3K (phosphoinositide 3-kinase)/mTOR (mammalian target of rapamycin) signaling pathway is activated by insulin, thereby up-regulating PKM2 expression through HIF1α-mediated transcription of the PKM gene (6, 9, 16, 32, 34).

A study performed by Panasyuk et al. proved that the expression of PKM2 is regulated by a nuclear hormone receptor, Peroxisome proliferator-activated receptor γ (PPARγ). The promoter region of PKM2 contains PPARγ response elements. AKT activation in PTEN-null fatty livers promotes the association of PPARγ to PPAR response elements (PPRE) and contributes to liver malignancies (33).

A previous study revealed that under physiological conditions, epidermal growth factor receptor (EFGR) up-regulated the transcription of PKM2 (35). Activation of EFGR induces phospholipase Cγ1-dependent protein kinase C (PKC)ε activation, which in turn ismonoubiquitinated at lysine 321 by E3 Ligase RING-finger protein (also known as TRIM41), which further interacts with C kinase 1 (34, 36). Monoubiquitinated PKCε combines with the ubiquitin-binding domain located in the zinc finger motif of IκB kinase (IKK)γ, and binding brings cytosolic IKK to the plasma membrane. PKCε phosphorylates IKKβ and, in turn, activates it. Activated IKKβ then phosphorylates IκB and revokes its repressive effect on v-rel avian reticuloendotheliosis viral oncogene homolog A (RelA), the p65 subunit of NF-κB, and allows it to translocate to the nucleus, where it directly combines with the PKM promoter and increases PKM2 expression (34, 36).

The expression of PKM2 is also subjected to post-translational modification, including phosphorylation, acetylation, succinylation, and oxidation. Acetylation of PKM2 at K305 decreases pyruvate kinase activity by suppressing the affinity for PEP (37). Also, acetylation of PKM2 at lysine-433 inhibits the activity of PKM2 by interfering with is allosteric activator, FBP, which prevents the tetramerization to the active enzyme form (21). PKM2 activity is down-regulated by phosphorylation at Y105 by Fibroblast growth factor receptor type 1 (FGFR1), which does not allow interaction of FBP withPKM2 (28). The phosphorylation induces the conversion of PKM2 from the tetramer to the dimer form, toward STAT3 phosphorylation (38). The PKM2 Y105F mutant expression reduces cell proliferation and tumorigenesisin HCC (28). JNK1 also phosphorylates PKM2 at Thr365, which increases PKM2 activity (39). PKM2 is succinylated at K498 and, upon succinylation, the activity of PKM2 increases (40). PKM2 activity is also down-regulated by oxidation of C358 by ROS or hypoxia, leading to switching of the flux of glucose into the pentose phosphate pathway and glycolytic biosynthesis to generate NADPH for ROS detoxification and tumor progression (24).

## Expression of PKM2 Under Normoxic and Hypoxic Conditions

Hasan et al. (41) demonstrated the expression of PKM2 in normoxic (20% O_2_) and hypoxic (0.1% O_2_) conditions in two prostate cancer cell lines, PC3 and LNCaP. They showed that that PKM2 mRNA was expressed at a higher level in the PC3 cell line than in LNCaP cells under normoxic conditions. Severe hypoxia significantly increased PKM2 mRNA expression in both cell lines (41). This suggested that, under hypoxic conditions, the expression of PKM2 is further promoted by HIF-1α activation.

HIF-1α is activated by inhibition of prolyl hydroxylase family (PHD1-3), which hydroxylates specific prolyl residues in the oxygen-dependent degradation domain of HIF-1α to increase its disruption. PHD3 hydroxylates specific proline residues on PKM2 in mild (1% O_2_) conditions but not in severe (0.1% O_2_) hypoxic conditions, thus favoring transactivation of HIF-1α (42).

## PKM2 and Cancer Metastasis

It is well known that many cancer cells grow under anaerobic conditions. However, unlike PKM1 or the tetramer PKM2, the dimer PKM2 synthesizes minimal (if any) ATP during the PEP-to-pyruvate conversion; thus, the net ATP gain for glycolysis is 4–2–2 = 0. It is already known that biochemical pathways are reprogrammed in many cancer cells, but it remains unclear how these cells generate sufficient energy with abnormal mitochondria. Tumor cells under hypoxic conditions, when the dimer PKM2 is active and OxPhos is suppressed, get energy from mitochondrial substrate-level phosphorylation (mSLP) (43). The literature suggests that when oxidative phosphorylation is impaired in tumor cells, the succinate-CoA ligase reaction in the tricarboxylic acid cycle can substantiate sufficient ATP through mitochondrial substrate-level phosphorylation (mSLP) for the growth of cancer cells. Production of high-energy phosphates would be supported by glutaminolysis through the sequential conversion of glutamine to glutamate and then to α-ketoglutarate, which is further converted to succinyl CoA, then to succinate. It is well-documented that tumors require a large amount of glutamine for survival and growth and that glutamine is a major energy source in cancer cells (44). Glutamine not only provides nitrogen for synthesis of nucleotides and NEAAs (nucleotides and non essential amino acid) but also provides a-ketoglutarate to serve as a precursor for ATP synthesis through substrate-level phosphorylation in the TCA cycle (43).

In order to replicate, a tumor cell requires lipids, proteins, carbohydrates, nucleotide precursors, and amino acid for processes like the formation of new membranes, protein glycosylation, DNA replication, RNA for new ribosomes, and the synthesis of other cellular molecules for cellular growth and proliferation (44, 45). ATP is required in the formation and assembly of these building blocks along with carbon and nitrogen (46). Significant expenditure of energy is required in the form of NADPH in *de novo* lipid synthesis. The oxidative PPP generates glycolytic intermediates like ribose-5-phosphate and phosphoribosyl pyrophosphate (PRPP) and reducing potential in the form of NADPH, which is needed for nucleotide biosynthesis (44). Glucose carbon is switched over from the normal glycolytic pathway to the anabolic metabolism used in serine and glycine biosynthesis, and dihydroxyacetone phosphate is used to make glycerol-3-phosphate for lipid biosynthesis (42, 44). In the final and rate-limiting step of glycolysis, the expression of PKM2 is up-regulated in lung, breast, cervix, kidney, bladder, papillary thyroid, colon, and prostate cancer (46, 47).

PKM2 protein switches between a highly active tetramer form and a low-activity dimer form in healthy tissue; however, it has distinct properties when acting as a dimer in tumor tissues. Numerous studies revealed that dimeric PKM2 had reduced catalytic activity, which participates in macromolecular biosynthesis, thus generating the materials needed for rapid cell proliferation (48). Apart from its role as pyruvate kinase, it functions as protein kinase and a transcriptional co-activator of many genes associated with tumor cell growth, metastasis, and cell death (11).

The relocation of PKM2 in the nucleus indicates that it plays a vital role in the nucleus. When PKM2 is present in the cytoplasm, it acts as a stable active tetramer, but when it is transported to the nucleus, it acts as a protein kinase, using PEP as a phosphate donor, and converts to the active dimeric form (49). In the nucleus, STAT3 is phosphorylated at tyrosine 705 by PKM2. This phosphorylation transcriptionally activates STAT3 and promotes transcription of MEK5 (MAP2K5) (49). Yang et al. demonstrated that upon activation of epidermal growth factor receptor (EGFR), PKM2 directly combines and phosphorylates histone H3 at threonine 11. This phosphorylation is essential for the removal of HDAC3 from the β catenin target gene CCND1 encoding for cyclin D1 and MYC promoter regions and histone H3 acetylation at Lysine 9 (38). All of the above-mentioned processes are vital for EGF-induced activation of cyclin D1 and c-Myc for cell proliferation and cell-cycle progression (50). Several studies demonstrated that upon EGF activation, PKM2 is transported to the nucleus by ERK2, where it binds to c-Src-phosphorylated Y333 of b-catenin, and both are recruited to the CCND1 promoter, leading to the removal of HDAC3 from the promoter region (38). The histone H3 acetylation at K9 and cyclin D1 expression lead to cancer cell proliferation and the development of brain tumorigenesis (50). PKM2 also interacts with Oct-4, a nuclear protein involved in cancer cell self-renewal and cell differentiation and HIF-1 as a co-activator in the nucleus, thereby enhancing their transcriptional activity (42, 51, 52).

Accumulating evidence indicates that tumor cells actively release a lot of exosomes, apoptotic bodies, or microparticles in order to communicate with the microenvironment and promote malignancy. These extracellular vehicles play a crucial role in the rapid proliferation of tumor cells (53, 54). PKM2 promotes exosomes release by phosphorylating synaptosome-associated protein 23, which in turn forms SNARE complex (55).

Growing pieces of evidence have shown that metastasis is specifically characterized by Epithelial-mesenchymal transition (EMT), resulting in inhibition of E-cadherin activity upon transportation of PKM2 into the nucleus (56). In the nucleus, PKM2 combines with TGF-β-induced factor homeobox 2 (TGIF2) and brings HDAC3 to the promoter of CDH1 gene, resulting in histone H3 deacetylation and reduced expression of the CDH1 gene encoding E-cadherin. As a result, transcription of E-cadherin is suppressed, leading to the loss of EMT, promoting cell invasion and metastasis (56, 57). Over expression of PKM2 facilitates nuclear translocation of STAT3, a transcriptional factor essential for PKM2-induced metastasis. The nuclear translocation and up-regulation of STAT3 are mediated by protein kinase activity of PKM2, which enhances migration and adhesion of cells in colon cancer (58).

## Pyruvate Kinase M2 in Tumor Angiogenesis

**Figure 3:**
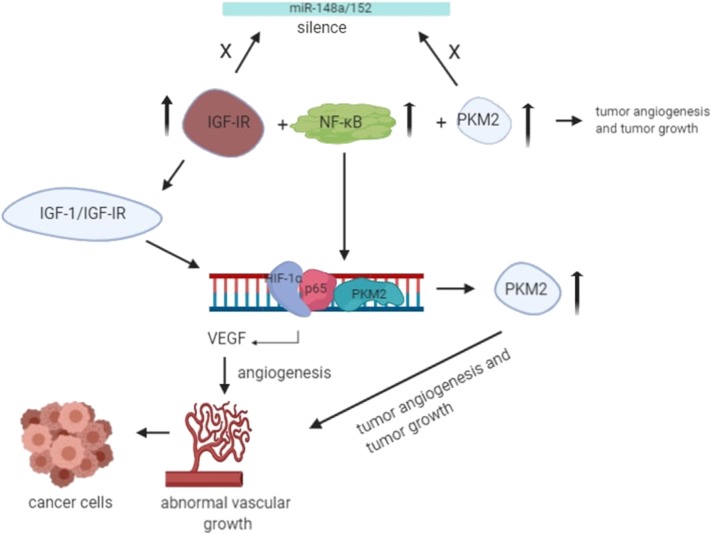
Under the hypoxic condition, translocation of PKM2 and p65 to the nucleus takes place. Upon interaction with PKM2, NF-_k_B subunit p65 activates the transcription of HIF-1α gene and its target gene, VEGF-A, in the nucleus. As a result, increased secretion of VEGF translates to increased blood vessel formation, contributing to tumor growth (55).

The angiogenesis of cancer cells has been a topic of research for decades. Several studies have been done to investigate the role of potential molecular drivers that help facilitate angiogenesis. Angiogenesis is the prevalent process shown by tumor cells; it is the generation of new blood vessels from existing blood vessels, which requires an extra supply of oxygen and nutrients. It is a very complicated process that involves vascular endothelial matrix disruption, migration, and proliferation of endothelial tissues (59). Tumor angiogenesis is initiated by dimer PKM2 in the blood, thereby increasing endothelial cell proliferation, migration, and cell-ECM adhesion, leading to the formation and growth of tumors (59, 60). It has become clear from the recent studies that IGF-IR activation is the most critical event involved in tumor angiogenesis, in which PKM2-mediated disruption of the NF-κB/miR-148a/152 feedback loop takes place, promoting tumor growth and angiogenesis (Figure 3) (30). Under hypoxic conditions, IGF-1/IGF-IR mediates the interaction ofHIF-1α with NF-κB subunit p65/RelA and PKM2 promoter, and the expression of PKM2 is also enhanced by miR-148a and miR-152 repression (61). The binding leads to nuclear translocation of PKM2, where it acts as protein kinase and interacts with other molecules to control the expression of vascular endothelial growth factor (VEGF), thereby promoting tumor angiogenesis (61).

However, additional studies are required to understand the mechanism of PKM2 interaction with the target molecules present on the surface of epithelial cells and the underlying mechanism that enables PKM2 to facilitate cell migration and adhesion to ECM in tumor growth.

## PKM2 as A Diagnostic Tool

Immunohistochemical staining of different tumors has revealed the presence of PKM2 in the tumor tissues. The distribution of PKM2 in primary tumors showed a heterogeneous distribution, and some areas consisted of highly stained PKM2, while other areas were weakly stained (62, 63). The expression of PKM2 was found to be enhanced in two different strains of hepatocellular carcinoma cell lines by using 2D gel electrophoresis followed by MALDI-TOF mass spectrometry (64). Immunohistological staining of blood taken from patients with colon cancer (12) and gastrointestinal (65), pancreatic, lung (66), ovarian (67), and renal cell carcinoma (RCC) (68) showed that PKM2 is released into the blood circulation. Several studies demonstrated that the expression of PKM2 is up-regulated in colorectal cancer patients (CRC) and inflammatory bowel disease and intrahepatic cholangiocarcinoma (ICC) tissues with high tumor cell necrosis, high angiogenesis, and more advanced stages. Therefore, PKM2 served as a prognostic marker for early detection of HCC (hepatocellular carcinoma) and ICC (69). PKM2 can also serve as a promising biomarker for chemosensitivity in patients with advanced non-small-cell lung cancer (NSCLC) receiving front-line platinum-based chemotherapy (70).

Moreover, enhanced expression of PKM2 is associated with the worst OS (overall survival) (70). Numerous studies have reported the PKM2 is highly expressed by regulating the expression of NEK2 in multiple myeloma, indicating that it could serve as a prognostic marker (2). In multiple myeloma patients, poor prognosis is associated with higher levels of PKM2 gene expression (71). High tumor cell metabolic activity and proliferative capacity are reflected by PKM2, so it serves as a non-organ-specific molecular marker (6). Additionally, some PKM2 is also released into neighboring tissues by necrosis of cancer cells and tumor cell renewal, which can be used in the early diagnosis of cancer. The PKM2 level in the circulating blood of patients may serve as a potential diagnostic marker in various carcinomas (67).

## PKM2 as A Potential Target for Cancer Therapy

As mentioned above, PKM2 plays a crucial role in cancer metabolism, thereby serving as a potential target for cancer therapy. Association of PKM2 with numerous external factors affects the metabolic activities of the cancer cell in many ways. Therefore, PKM2 can serve as a target for cancer treatment. There are several targets that are under preclinical and clinical trials (72, 73). PKM2 is explicitly expressed in tumor cells, so down-regulating the expression of PKM2 may suppress the growth of tumor cells. Several studies have shown that when shRNA and miRNA interfere with the expression of PKM2, both lead to the death of cancer cells, decreased metabolic activity, and reduced tumorigenesis (3, 74). Several other studies have revealed that when shRNA interferes with PKM2 expression, the sensitivity of tumor cells to certain drugs like docetaxel and cisplatinis increased, thereby promoting the death of tumor tissues and reduced tumorigenesis (75, 76). There are many small molecule inhibitors and hormones that inhibit cell proliferation by targeting PKM2 (77–79). The inhibitors, namely shikonin and its analogs, flavonoid derivatives, and 2,3-dithiocarbamate substituted naphthoquinones bind to the allosteric site of PKM2, which leads to reduced glycolysis in cancer cells (77, 80–82).

Additionally, there are pieces of evidence that show that siRNA-mediated targeting of PKM2 at the mRNA level leads to caspase-mediated apoptosis in cancer cells (83). A significant chunk of data reveals that inhibition of PKM2 could improve the sensitivity to drugs in cancer cells. For example, the interaction of PKM2 with CD44 suppresses the activity of PKM2 by increasing its phosphorylation at threonine 105, with high production of ROS, thereby increasing cisplatin-sensitivity in colorectal cancer cells. Moreover, ablation of CD44 rewires aerobic glycolysis into the TCA cycle, and production of ROS is increased, which in turn increases the cisplatin-sensitivity of colorectal cancer cells (CRC) (84). Therefore, cisplatin resistance can be easily overcome in many cancer cells by inhibiting the expression of PKM2 where cisplatin is used. In patients with breast cancer, the expression ofPKM2 is related to *in vitro* chemosensitivity to epirubicin (EPI) and 5-fluorouracil (5-fu). The relationship between PKM2 expression and the sensitivity of the patient to EPI and 5-fu has been demonstrated by specific experiments, which suggested that patients that show increased PKM2 expression should be considered for EPI-based treatment or EPI in combination with 5-fu chemotherapy to get an effective prognosis (85).

Besides, there are several peptide aptamers that inhibit PKM2 and not PKM1, thereby decreasing tumor cell proliferation, growth, and size under conditions of metabolic reprogramming in favor of cancer cells (86). Most of the cancer cells show high glutaminolysis capacity, which serves as another source of energy for these cells. Glutamine is the most abundant amino acid found in the blood and is involved in many aspects of cancer metabolism (87). Cancer cells utilize glutamine for energy production and also as a precursor for rapid biomass production for fast-growing cancer cells (88).

Therefore, PKM2 inhibition does not remarkably decrease the growth of tumor cells. Quinolone sulfonamide is the PKM2 activator that promotes the conversion of PKM2 from dimerto tetramer. This results in a decreased synthesis of the glycolytic intermediates used as biosynthetic precursors by cancer cells. These activators bind to a pocket away from the binding site of FBP, resulting in a rerouting of glycolytic intermediates away from the serine biosynthetic pathway producing serine for continued cell growth in cancer (89). Additionally, PKM2 activators reduce the growth of xenograft tumors like aggressive lung adenocarcinoma (90).

There are several PKM2 activators and inhibitors that are in preclinical and clinical trials, and the results of these studies show that these inhibitors and activators could be promising anti-cancer drugs.

## Concluding Remarks

This review mainly deals with the metabolic functions of PKM2, its regulation, and its role as a therapeutic agent. It has become clear from the past research that PKM2 is involved in cancer proliferation through metabolic reprogramming. PKM2 is a multifaceted protein that appears to play a similar role in diseases like diabetic nephropathy (91) as well as cancer. The research into the expression of PKM2 in tumor cells and other proliferating cells focuses on the role of PKM2 in mediating cancer cell metabolism. Numerous proteins have been identified as a substrate for PKM2, and many of them are involved in the regulation of cancer cell growth. Certain studies have reported that overexpression of PKM2 might act as a potential biomarker for specific types of cancer. However, it is not clearly understood how the expression of PKM2 changes the reaction of the cell upon the activation of the growth factor. The interaction of PKM2 with growth factor shows that the regulation is integrated. Intracellular ROS accumulation is also prevented by PKM2, thereby enabling the subsistence of cancer cells under oxidative stress.

In conclusion, PKM2 is involved in both glycolytic and non-glycolytic pathways and is instrumental in the malignancy of tumor cells, suggesting that it could act as a remarkable therapeutic target. However, the intracellular event brought about by PKM2 is far more complicated than previously hypothesized. Therefore, further studies are required in order to make PKM2 a fruitful target for cancer therapy, and there is a need to qualitatively and quantitatively measure the level of PKM2 in cancer patients in order to make PKM2 a successful drug target.

## Ethics Statement

Ethical clearance was obtained from the institutional ethical committee of our institution prior to starting the study (reference no: ECR/Bhu/Inst/UP/2013/Re-registration-2017 dt. 31.01.2017).

## Author Contributions

KZ drafted the review article. TD helped in revising it critically for important intellectual content. A helped in design and drafting of the article. SM gave final approval of the review to be published. UP gave feedback and suggestions for the article.

### Conflict of Interest

The authors declare that the research was conducted in the absence of any commercial or financial relationships that could be construed as a potential conflict of interest.
